# Size matters: When resource accessibility by ecosystem engineering elicits wood‐boring beetle demographic responses

**DOI:** 10.1002/ece3.7079

**Published:** 2020-12-07

**Authors:** Alexandre Mourant, Nicolas Lecomte, Gaétan Moreau

**Affiliations:** ^1^ Département de biologie Université de Moncton Moncton NB Canada; ^2^ Canada Research Chair in Polar and Boreal Ecology Université de Moncton Moncton NB Canada

**Keywords:** Canadian beaver, Coleoptera, food web, resource pulse, Saproxylic beetles

## Abstract

Episodic natural disturbances play a key role in ecosystem renewal, and ecological engineering could do so by transforming resource accessibility. While such coupling creates nontrophic and lasting interactions between resource consumers and ecosystem engineers, it is unclear how large the disturbance must be to sustain such coupling. Natural disturbances that occur from the ecological engineering by the Canadian beaver (*Castor canadensis*) modulate deadwood dynamics in many forest ecosystems. Relying on such episodes of fresh woody debris, primary wood‐boring beetles, organisms that dig tunnels into those debris for reproduction, act as important deadwood decomposers in the ecosystem. Here, we investigate how the age and size of beaver disturbances act as predictors for primary wood‐boring beetle abundance and species richness around beaver‐altered habitat patches. To do so, we sampled beetles around 16 beaver‐disturbed and unaltered watercourses within the Kouchibouguac National Park (Canada) and modeled beetle demographic responses to site conditions and their physical characteristics, distance from the watercourse, deadwood biomass, and the geographical location of the sites. Our results indicate that the size of the disturbance is positively associated with beetle abundance, which highlights unique deadwood dynamics inherent to large beaver ponds. The role of beavers in forest ecosystems by reaching multiple taxa at multiple spatiotemporal scales further exemplifies the need to study nontrophic interactions and their complex consequences in ecosystem management.

## Graphical abstract

The size of the disturbance created by beavers was positively associated with primary wood‐boring beetle abundance around beaver‐altered habitat patches. This shows that ecological engineering transforms resource accessibility, creating nontrophic and lasting interactions between resource consumers and ecosystem engineers.

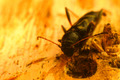

## 1 INTRODUCTION

Disturbances represent an important component of ecosystem dynamics (Bengtsson et al., 2003). Episodic natural disturbances, for instance, are known to play a key role at different scales of magnitude in ecosystem renewal (Holling, 1986), spatial heterogeneity (Bishop et al., 2009), and resource accessibility (Johansson, 2006; Yang et al., 2008). Disturbances can be driven by abiotic (e.g., strong winds, intense rainfall, fire) or biotic events (e.g., insect outbreaks, seed or fruit mast events). Such disturbances will be referred to as a resource pulse when a large quantity of ephemeral resources become available to consumers in a short time period following the disturbance (Yang et al., 2008, 2010). Resource pulses vary in magnitude, both spatially and temporally, and generally induce a strong response from primary consumers located one trophic level above the pulsed resource. Primary consumers are expected to be the first organisms to respond to the pulse (e.g., Yang et al., 2010), and have the potential to produce repercussions throughout the food web (Ostfeld & Keesing, 2000).

Among the natural disturbances that could elicit a response from primary consumers is the sudden resource accessibility caused by ecosystem engineering. Ecosystem engineers are organisms that rearrange physical proprieties of ecosystems throughout their activities, which modify accessibility to nutrients for several other species (Jones et al., 1997). As a result, disturbances by ecosystem engineers modulate the links and nodes of food webs, thus indirectly affecting consumers (Kéfi et al., 2012; Sanders et al., 2014). It is thus fair to expect that ecosystem engineers might generate resource pulses by suddenly releasing nutrients that have accumulated over time in the engineered system. Engineered resource pulses can be expected to have disproportionally large impacts on ecosystems relative to their inherent organismal and demographic characteristics, impacting resident species for a long time, even longer than the engineer's habitat utilization (e.g., Hastings et al., 2007). However, the association between the spatial scale of the disturbance and the characteristics of the disturbance such as its longevity in the system and the magnitude of its impact on consumers remains largely understudied.

A well‐documented example of these effects comes from the damming of streams and foraging performed by the Canadian beaver (*Castor canadensis*) (e.g., Levanoni, 2016; Stokland et al., 2012). Following beaver dam construction, trees affected by the flooding die from anoxia (Snodgrass, 1997) and neighboring preferred trees and branches (mainly *Populus tremuloides* and species of *Salix* L.) can be cut and displaced by the beaver (Gallant et al., 2004). Branches of a diameter that is not valued by the beaver, as well as other tree species (e.g., *Alnus* spp.), can also be displaced for the construction of the dam (Barnes & Mallik, 1996). This results in a fast and localized accumulation of horizontal and vertical fresh woody debris in the disturbed area, which can, in turn, attract primary wood decomposers (Saarenmaa, 1978; Thompson et al., 2016). After beaver colonization, beaver ponds remain occupied for approximately 4 years (range: 1–20), and will later be abandoned by beavers (Hastings et al., 2007; Nummi & Kuuluvainen, 2013; Warren, 1932). These abandoned ponds can eventually be recolonized when resources are again adequate for beavers (i.e., about every 10 years in boreal forests). Alternatively, these ponds can dry out and transform into meadows that can last up to 70 years (Hastings et al., 2007; Hyvönen & Nummi, 2008).

**FIGURE 1:**
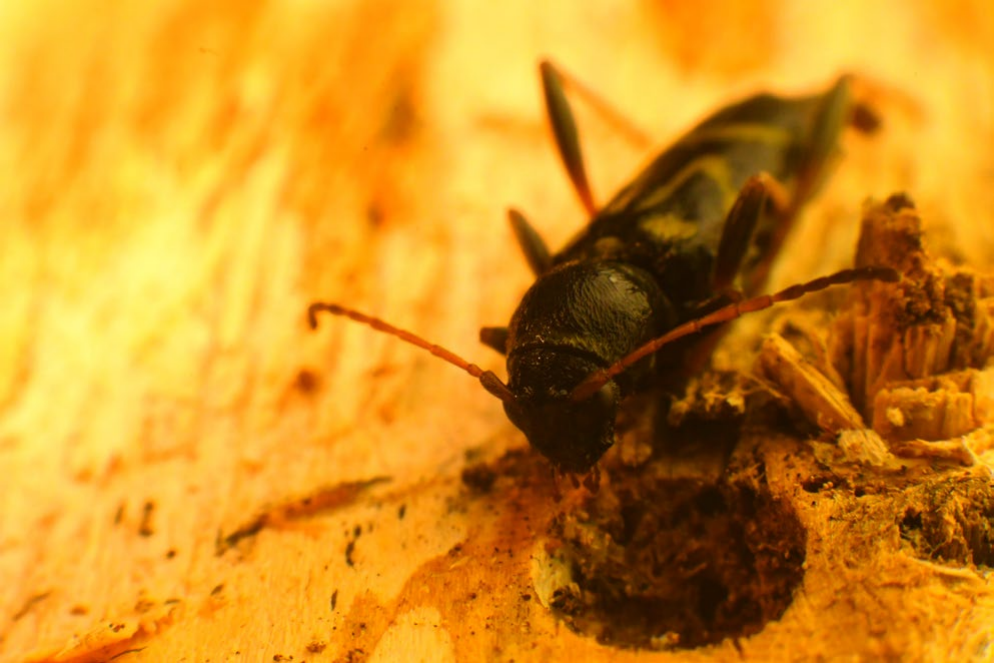
*Clytus ruricola* (Olivier, 1795), a species of beetle in the family Cerambycidae that was frequently recovered during the study, next to an emergence hole

Wood‐boring insects (Figure 1), which are mainly from the Order Coleoptera (i.e., beetles), are known as primary deadwood consumers (Nadeau, Thibault, et al., 2015). Since members from this taxon generally prefer fresh woody debris, primary wood‐boring beetles have a strong potential to respond to a deadwood resource pulse (e.g., Gandiaga & Moreau, 2019; Thibault & Moreau, 2016b). These insects also demonstrate species‐specific affinity among resources (Grove, 2002) and a general preference for sun‐exposed debris (Lindhe et al., 2005; Simila et al., 2002). In addition, wood‐boring beetles display a strong capacity for olfactory detection of volatile chemicals released by stressed trees (Erbilgin et al., 2007) and an efficient communication systems through pheromones triggering aggregative response (Franceschi et al., 2005). Such olfactory systems are combined with efficient flight dispersal abilities (e.g., in extreme cases, up to 171 km for anemochorously displaced bark beetles in Nilssen, 1984). Also, since riparian lowlands exhibit the potential to harbor large trees, beaver impoundments can generate large diameter snags, which is a generally favored resource for a large spectrum of wood‐boring beetles (Grove, 2002; Pollock et al., 1995). These organisms are also of importance from a conservation perspective because their tunneling activity speeds up wood decomposition by physically and chemically breaking down deadwood, thereby facilitating fungi and bacteria colonization deeper in the debris (Esseen et al., 1997; Harmon et al., 1986; Siitonen, 2001).

Here, we investigated the response of wood‐boring beetle feeding guilds to ecosystem engineering by beavers. The hypotheses and prediction are described in Table 1. To test these four complementary hypotheses, we designed an insect trapping study and recorded dead wood biomass with standardized protocols in an area rich in beaver sites over a two‐year period. To our knowledge, this is the first study to assess primary wood‐boring beetle activity surrounding beaver ponds. The only similar study that we found is from Saarenmaa (1978), in Finland, who sampled a selection of 20 bark beetle species in a beaver‐flooded Norway spruce stand. This has led many authors to call for studies on the potential interaction between beavers and saproxylic organisms, and for work on the aquatic and terrestrial ecological linkages inherent to beaver disturbances (e.g., Ecke et al., 2017; Nummi & Kuuluvainen, 2013; Thompson et al., 2016).

**TABLE 1:** Hypotheses and predictions

Hypothesis	Prediction	Accepted/Refuted	References
(H1) Ecosystem engineering by beavers modifies the habitat in a way that is favorable for primary wood‐boring beetles because it renders important quantities of fresh deadwood available to colonization	(P1) Increasing abundances of wood‐boring beetles with disturbance perimeter	A	Thompson et al. (2016)
(H2) Deadwood resources generated throughout beaver engineering will lose their attractancy for primary wood‐boring beetles as they age because the physical characteristics of the deadwood will deteriorate over time	(P2) Decreasing abundances of wood‐boring beetles with increasing pond age	R	Lee et al. (2014), Nadeau, Majka, et al. (2015), Nadeau, Thibault, et al. (2015), Vanderwel et al. (2006)
(H3) Ecosystem engineering from beavers creates a sudden input of deadwood with a diverse spatial orientation, essence and diameter that can attract a surge of species richness of primary wood‐boring beetles	(P3) Increasing species richness of wood‐boring beetles with disturbance perimeter	R	Thompson et al. (2016)
(H4) Deadwood microhabitats harbored in beaver ponds will diminish in quality along with the age of the disturbance because of the reducing physical attractancy of the resource for primary wood‐boring beetles	(P4) Decreasing species richness of wood‐boring beetles with increasing pond age	R	Nadeau, Majka, et al. (2015), Nadeau, Thibault, et al. (2015)

## 2 MATERIAL AND METHODS

### 2.1 Study sites

The study was conducted in the Acadian forest biome at Kouchibouguac National Park (lat. 46°48′N, long. 64°54′W), a 238‐km^2^ federal park located in eastern New Brunswick, Canada. We used 16 sites previously identified as beaver‐engineered (BEA), where watercourses encompassed beaver activity material (i.e., beaver dam, pond and/or meadow). We determined the age (in years; 12–44 years) and the perimeter (in m; 135–889 m) of the disturbed area through aerial photography. We paired BEA sites with control sites (CON), which each included a watercourse without any sign of beaver activity within 100‐m radius from the CON periphery. Hereafter, we will use the term “site condition” as a general concept encompassing both BEA and CON.

At each site, we ran a 50‐m transect oriented perpendicularly to the watercourse. For BEA, each transect began at the limit of the flooded area. The transects for meadows began at the end of habitat patches linked to ancient flooding (Johnston, 2012). Transects in BEA and CON intersected the same type of habitats since we used beaver ponds with a sharp perturbation edge, preventing any bias of wetlands (not directly linked to beaver activity) buffering the beaver‐modified habitat.

### 2.2 Insect data

Flight‐intercept traps (FIT) (e.g., Nadeau et al., 2015) were installed at 5, 20, and 50 m along the transects. Each trap was composed of two perpendicularly inserted 30.5 × 61 cm acrylic sheets under which a styrene funnel was attached. A plastic cup was placed at the bottom of every funnel and contained a mixture of 70% ethanol and a drop of dishwashing soap. Two clothespins were pinned on the base of an acrylic sheet to stabilize the FIT, and a round styrene cover was placed over the FIT to prevent debris and rain from accumulating in the collecting cup. Each trap was suspended at 1.6 m above ground, from the center of the acrylic sheets, on a rope attached to two trees that were less than 4 m apart. Placing the traps between the trees rather than against the trunk of a given tree species allowed for the sampling of all saproxylic beetles and not only that of the beetles attracted to a given host species. While window traps baited with ethanol are more efficient to trap saproxylic beetles than unbaited traps in terms of abundance and richness (Bouget et al., 2009), previous work has shown that these traps are highly sensible to detect localized changes in the resource (Gandiaga et al., 2018; Thibault & Moreau, 2016b) and that their efficiency does not vary with forest stand type (Bouget et al., 2009).

From the beginning of June to the end of August in 2015 and 2016, traps were emptied bimonthly to recover wood‐boring beetles. We only selected beetle taxa known to actively bore into fresh dead wood material (i.e., Ptinidae, Lymexylidae, Cerambycidae, and Buprestidae families, and Scolytinae, Conoderinae, and Cossoninae subfamilies; Arnett et al., 2012; Jones et al., 2007; Kirkendall et al., 2015). All specimens were identified to the species level using current taxonomic keys (see Appendix) and voucher specimens from the collection of Université de Moncton. Beetles were also classified in three trophic categories, being coniferous borers, deciduous borers or generalist borers (both coniferous and deciduous borers) (see Appendix).

### 2.3 Deadwood biomass measurements

We quantified the deadwood biomass at our study sites to account for the effects of available resources on beetle activity. We established a 50‐m^2^ quadrat (5 m × 10 m) next to each trap, on the right‐hand side of the transect from the watercourse perspective. We measured the diameter and volume, and determined the type (deciduous or coniferous) of all horizontal (i.e., logs, stumps, and branches) or vertical (i.e., snags) woody debris over 2 cm in diameter following the method described in Thibault and Moreau (2016a). Using this method, debris were ranked from 1 (fresh) to 5 (fully rotten horizontal debris or snags smaller than 2 m, showing an advanced stage of decay).

### 2.4 Statistical analyses

**FIGURE 2:**
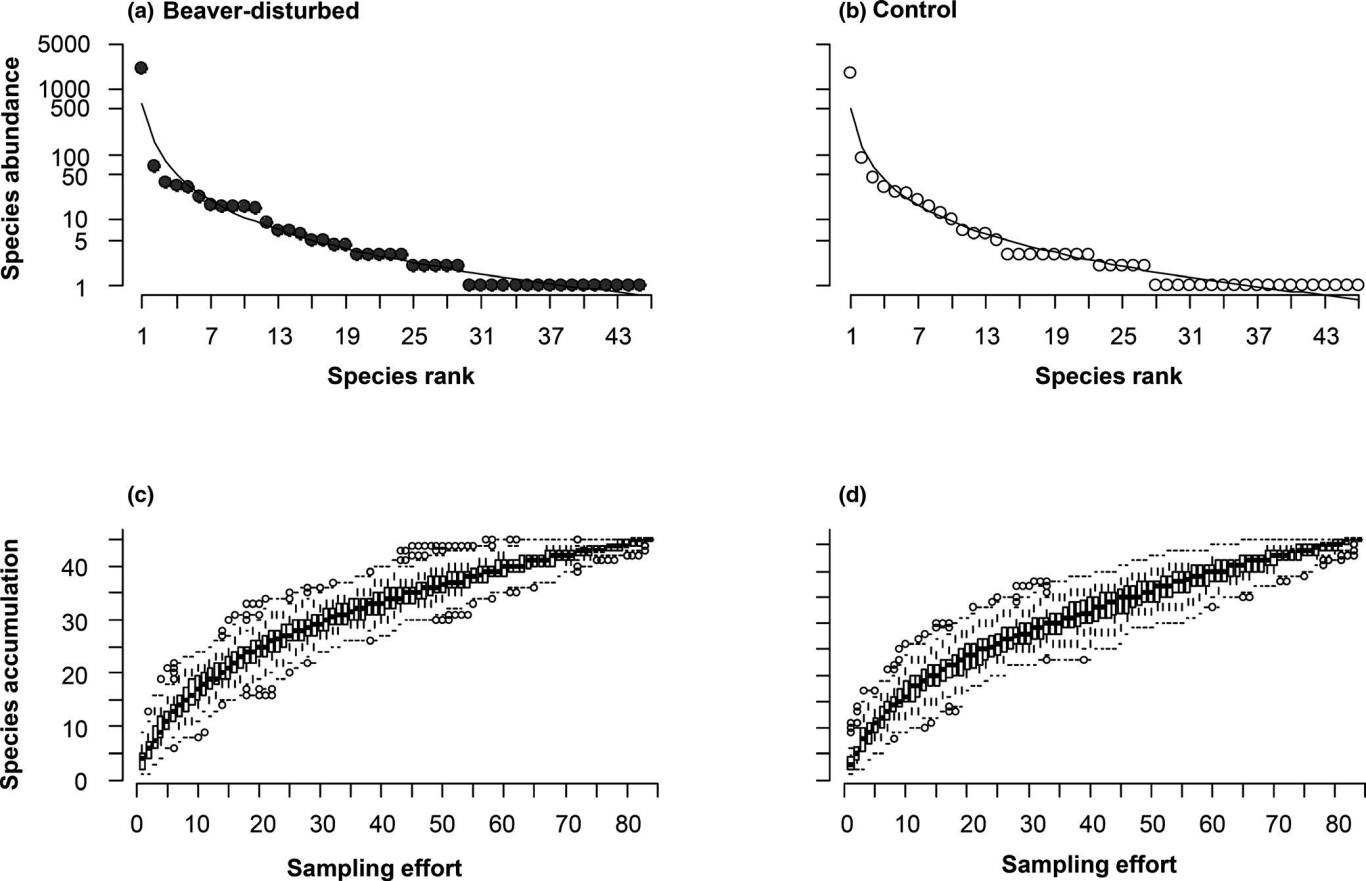
Rank–abundance (a, b) and rarefaction curves (c, d) of wood‐boring beetle communities in beaver‐disturbed (a, c) and control (b, d) sites in the Kouchibouguac National Park, Canada, in 2015–2016. Species accumulation is represented as a function of the sampling effort, while species abundances are represented as a function of species ranks. The *y*‐axis for both former graphs is in a logarithmic scale, and for both site conditions, *Anisandrus sayi* occupies the first rank

Statistical analyses were performed using R version 3.4.2 (R Core Team, 2018). Care was taken to ensure that the postulates of all analyses were met. To explore beetle community parameters such as composition and individual rarity, we plotted a rarefaction curve and a rank abundance curve for both site conditions (Figure 2). Based on an exploratory principal component analysis carried out to detect correlated variables, we pooled the volume of all debris (snags and logs) of the same essence (coniferous or deciduous) for fresh debris (classes 1–2) and old debris (classes 3–4–5), creating four variables describing deadwood biomass.

We used generalized additive mixed models (GAMMs) to examine the effect of ordinal numeric variables (i.e., distance from the watercourse, perimeter of the disturbance, disturbance age, site coordinates, class 1–2 hardwood/softwood biomass, and class 3–4–5 hardwood/softwood biomass) and a categorical variable (i.e., site condition), on five dependent variables (i.e., *Anisandrus sayi* abundance, coniferous‐borer abundance/richness, deciduous‐borer abundance/richness) (Table 2). None of the independent variables were strongly autocorrelated (−0.70 < *r* < 0.70) and for all analyses, we used the complete models. *Anisandrus sayi* abundance was analyzed separately from other deciduous borers because of its sheer abundance compared with other species in this study (see Results) and other work carried out in New Brunswick, Canada (Gandiaga & Moreau, 2019). Ordinal numeric variables were considered as smooth terms, and categorical variables were considered as parametric terms. The sampling year (i.e., 2015 and 2016) and the block formed by each pair of BEA‐CON sites were included as random effects in all models. The dependent variables were fitted using a Poisson distribution. A multivariate regression tree (MRT) and a MRT‐based ordination were carried out but are not presented herein because they produced little clustering as beaver disturbance mostly affects beetle abundance (see Results).

**TABLE 2:** *F*‐values of smooth and parametric terms of GAMMs analyzing the abundance or richness of coniferous or deciduous borers and *Anisandrus sayi* abundance within Kouchibouguac National Park, Canada, in 2015–2016

Term	Type	Abundance			Species richness	
		*Anisandrus sayi*	Other deciduous‐borers	Coniferous‐borers	Deciduous‐borers	Coniferous‐borers
Class 1–2 softwood biomass	Smooth term	8.83**	17.78**	10.40**	0.25	3.59
Class 3–4–5 softwood biomass	Smooth term	3.54*	7.22**	20.71**	2.05	3.98*
Class 1–2 hardwood biomass	Smooth term	24.00**	0.14	8.23**	0.05	8.70**
Class 3–4–5 hardwood biomass	Smooth term	17.22**	1.00	4.21*	0.41	0.02
Perimeter	Smooth term	13.81**	17.53**	17.18**	0.01	2.63
Age of the disturbance	Smooth term	4.32*	3.97*	0.34	0.32	3.12
Distance in beaver‐modified sites	Smooth term	5.19*	8.67**	5.57**	2.07	0.11
Distance in control sites	Smooth term	3.75*	0.80	19.48**	0.07	3.32*
GPS coordinates	Smooth term	29.95**	8.05**	12.49**	2.86*	4.72**
Site condition	Parametric term	4.12*	9.75**	3.36	0.05	2.67
	*r* ^2^	0.45	0.23	0.36	0.07	0.24

Note

Dark gray and light gray areas indicate positive and negative effects, respectively.

* 0.05 ≥ *p* ≥ 0.01.

** *p* < 0.01.

**FIGURE 3:**
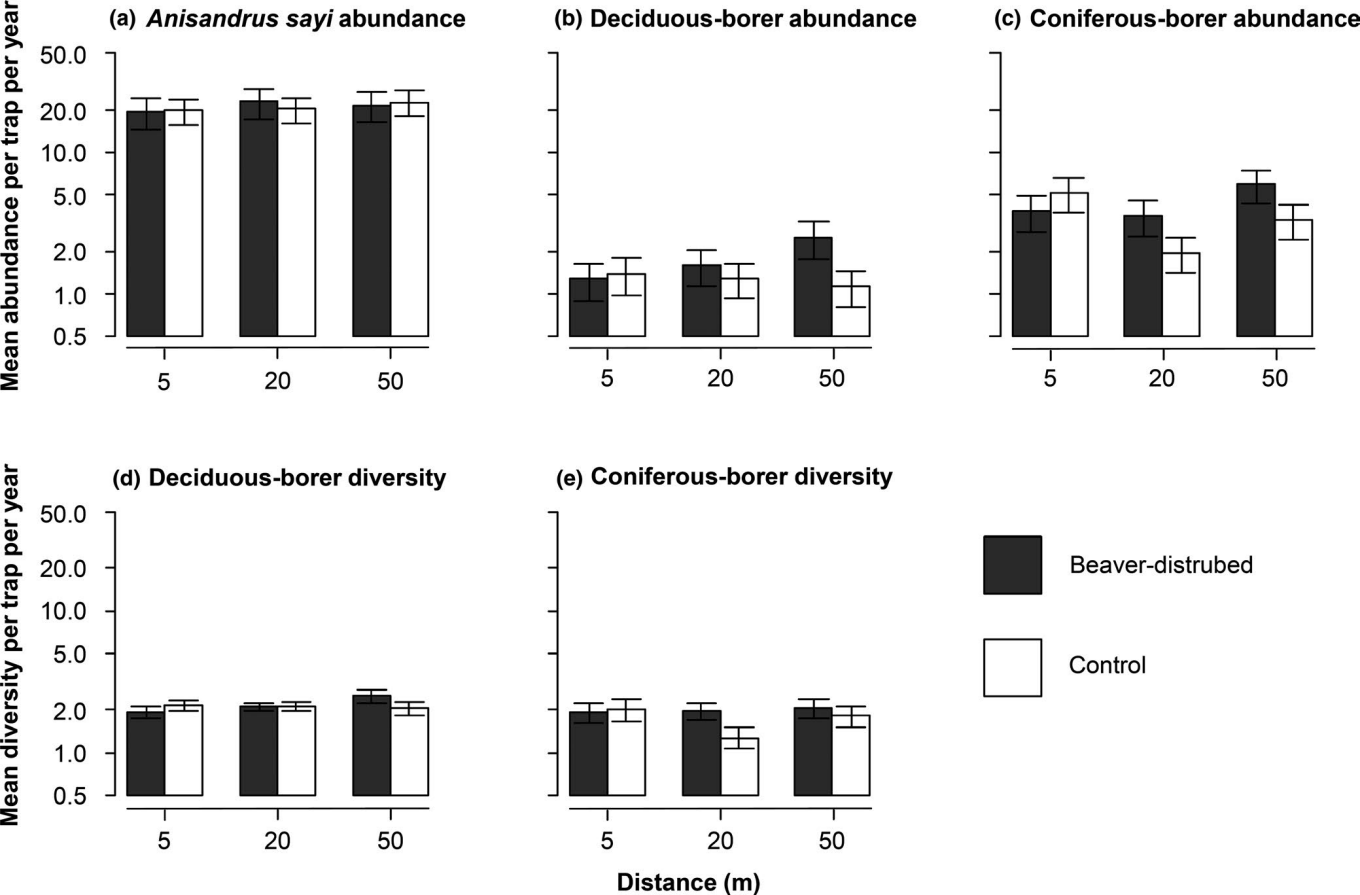
GAMM predictions displaying (a) *Anisandrus sayi* abundance, (b) deciduous‐boring beetle abundance, (c) coniferous‐boring beetle abundance, (d) deciduous‐boring beetle species richness, and (e) coniferous‐boring beetle species richness in the Kouchibouguac National Park, Canada, in 2015–2016. Predicted values per trap per year are represented as a function of the distance, with black bars representing beaver‐disturbed sites and white bars representing control sites. The *y*‐axis for all graphs is in a logarithmic scale. Other variables were kept constant

**FIGURE 4:**
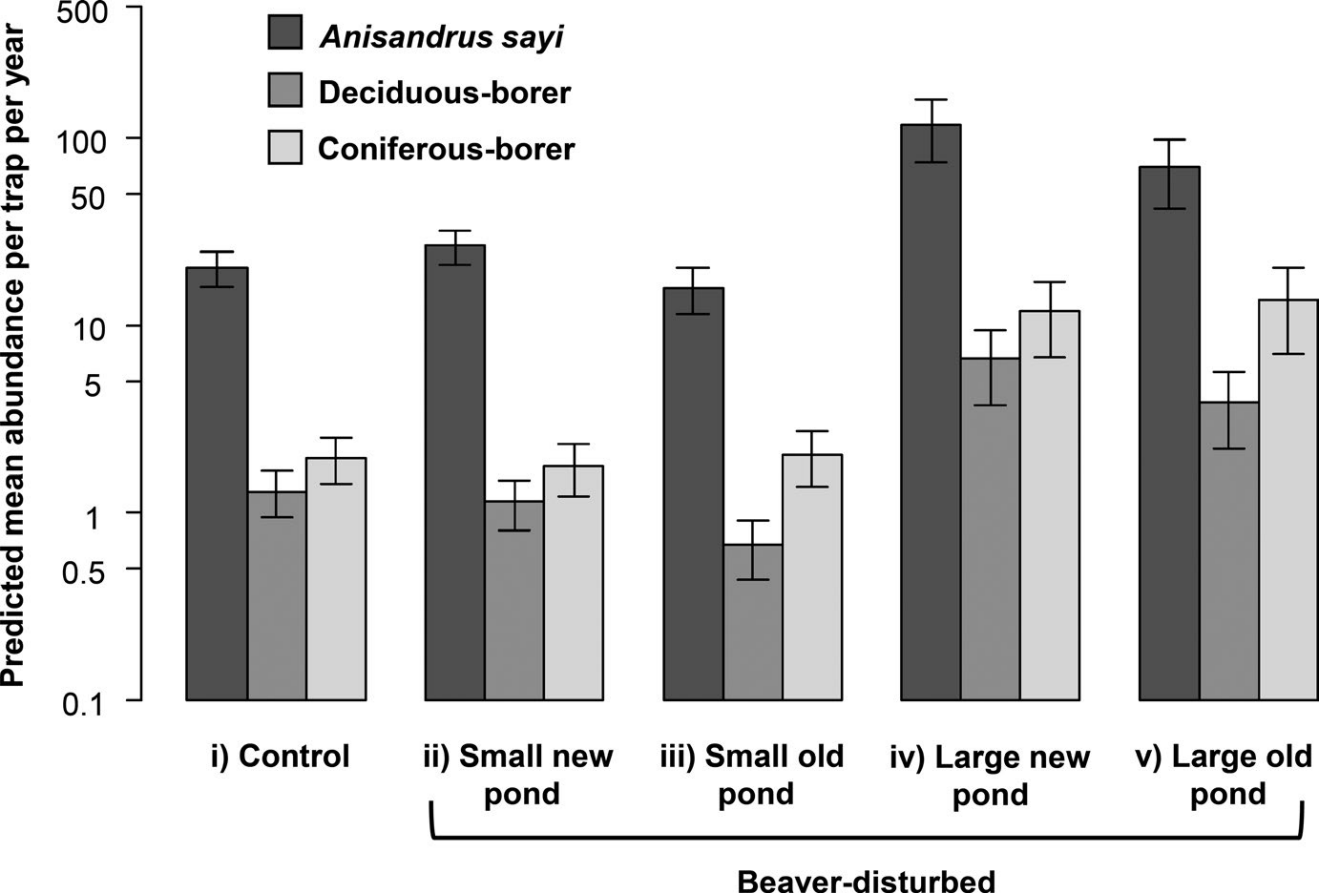
GAMM predictions displaying *Anisandrus sayi* (dark gray), deciduous‐boring (gray) and coniferous‐boring (light gray) beetle abundances by trophic category in the Kouchibouguac National Park, Canada, in 2015–2016. Predicted values per trap per year are represented on a logarithmic scale as a function of different scenarios, which are (i) control, (ii) small younger pond, (iii) small older pond, (iv) large younger pond, and (v) large older pond sites. Other variables were kept constant

To determine the effect of conditions while controlling for the other variables, we plotted predictions from GAMMs for each site condition (Figure 3) with all deadwood biomass variables set on the mean volume documented during the study. For these predictions, the perimeter and age of the disturbance were set at the mean value for BEA, at zero for CON, and the coordinates used corresponded to an area where the additive effect of the periphery and age of the disturbance was zero. We also ran a second set of statistical predictions (Figure 4) for beetle abundance in which for BEA, we fixed the perimeter and age of the disturbance at both their maximal and minimal values, for every combination possible. In this second set of predictions, we fixed deadwood biomass variables on their mean values, and the distance from the watercourse at 20 m.

## 3 RESULTS

4,524 primary wood‐boring beetle from 61 species were collected during this study (Table A1). Of the 61 species, 23 were singletons (i.e., collected a single time) and one, *A. sayi*, was collected 3,814 times.

### 3.1 Community structure

Rarefaction curves for both BEA and CON were close to a plateau, suggesting that more species would probably be found if the study had lasted longer than 2 years (Figure 2c,d). Nevertheless, rarefaction curves for both site conditions exhibited the same general shape, suggesting that species accumulated slowly but at a similar rate in both communities (Figure 2c,d).

As with rarefaction, rank–abundance curves for both site conditions exhibited the same general pattern, which indicates that the proportion allocated to individual species in beetle communities was similar between site conditions (Figure 2a,b). Another key component of these communities is that both were largely dominated by *A. sayi*, which was 30 and 20 times more abundant than the second‐ranked species in BEA and CON, respectively (Figure 2a,b). More than half of the species in each site condition were recovered less than five times, and singletons account for 36% and 41% of the communities in BEA and CON, respectively (Figure 2a,b; Appendix), indicating that communities in both site conditions largely comprised infrequent and rarely collected species.

### 3.2 Species abundance

Since much of beetle abundances were positively affected by the perimeter and the age of the disturbance, we further analyzed these parameters with statistical predictions. The abundance of the three main wood borer taxa (i.e., *A. sayi*, other deciduous‐boring beetles and coniferous‐boring beetles) was affected to different extents by the studied variables. For *A. sayi*, most of our study variables had a positive impact on abundance as revealed by a GAMM explaining 45% of the variability (Table 2). Nevertheless, although site condition and distance to the watercourse had an effect on *A. sayi* abundance (Table 2), GAMM predictions indicated that these effects were relatively minor (Figure 3a). For other deciduous‐boring beetles, softwood biomass, disturbance perimeter, age of the disturbance, and distance to the watercourse in BEA had a positive effect on their abundance, as revealed by a second GAMM explaining 23% of the variability (Table 2). Site condition also influenced deciduous borers (Table 2), with GAMM predictions showing that abundance was increasing with the distance in BEA but not in CON (Figure 3b). For coniferous‐boring beetles, softwood biomass, old hardwood biomass, disturbance perimeter, and distance in BEA had a positive effect on abundance, as revealed by a third GAMM explaining 36% of the variability (Table 2). Hardwood biomass and distance in CON affected the latter abundance negatively (Table 2). GAMM predictions indicated that distance to the watercourse was having a positive and negative effect on coniferous‐boring beetle abundance in BEA and CON, respectively (Figure 3c).

### 3.3 Species richness

The richness in deciduous‐boring beetles and coniferous‐boring beetles was affected by the studied variables but to a lower extent than abundance. For deciduous‐boring beetle, only the GPS coordinates had an effect on richness, as revealed by a GAMM explaining 7% of the variability (Table 2; see Figure 3d for GAMM predictions). For coniferous‐boring beetles, old softwood biomass had a positive effect on richness, while fresh hardwood biomass and distance in CON had a negative effect, as revealed by a second GAMM explaining 24% of the variability (Table 2). Prediction from the GAMM showed a generally negative effect of distance to the watercourse on CON, with a higher predicted richness in BEA compared with CON at 20 m (Figure 3e). However, no effects of disturbance perimeter or age were detected, so we reject P3 and P4 (Table 1), without the need to run statistical predictions regarding these variables.

### 3.4 General trends

Our analysis indicates that age and size of the disturbance affected the complex of wood‐boring beetle, mostly in terms of abundance, but that these effects can be blurred by deadwood biomass. Thus, to test P_1_ and P_2_, we used the GAMMs developed above to make predictions about beetle communities in small, large, younger, and older beaver‐disturbed sites, while maintaining the deadwood biomass to an averaged value (Figure 4). These predictions showed that pond dimensions affect wood‐boring beetle communities. Smaller ponds, irrespective of their age, showed no difference in predicted mean abundance for all feeding guilds compared with control sites, while younger and older large ponds exhibited ca. 6 and 4 times more beetles than control sites, respectively (Figure 4).

## 4 DISCUSSION

This study demonstrated a positive association between beaver disturbances and wood‐boring beetle activity. The main variable that positively affected primary wood‐boring beetle abundance was the perimeter of the disturbed area, which supports P_1_ and validates H_1_. Indeed, the perimeter variable was one of the most explicative variables throughout our analyses of wood‐boring beetle abundance (Table 2; Figure 4). Larger beaver ponds have a higher probability to harbor more deadwood biomass than small ones. Such result could reflect a higher beetle activity directly driven by a proportional response to their resources provided by the disturbed area (e.g., Stokland et al., 2012; Thompson et al., 2016). By contrast, the age since the initial disturbance was not linked with higher beetle abundance nor species richness. In addition, the perimeter of the disturbance had little to no effect on species richness. Hence, we could not find evidence to support P_2_, P_3_, and P_4_, thus leading us to reject H_2_, H_3_, and H_4_.

Although we did not find a generalized response for decreasing beetle abundance with increasing pond age to validate P_2_, some feeding guild‐specific effects occurred. For instance, predicted *A. sayi* abundance in small ponds (Figure 4ii,iii) was higher for younger ponds compared with old ones, which highlights the affinity of this species for fresher debris. Primary wood‐boring beetles are attracted to fresh debris, and when they do leave them, the debris can then be colonized by later‐successional organisms (Abrahamsson et al., 2009; Hammond et al., 2001; Lee et al., 2014). In this context, we might reject P_2_ because beaver ponds were too old (12–44 years old) to effectively assess the early successional phase of feeding guilds. Accordingly, the rejection of P_3_ and P_4_ could be explained by three reasons. First, the effect of beaver disturbances on primary wood‐boring beetle species richness could mostly be noticeable at the first flood, when the initial heterogenic resource pulse is created (e.g., Levanoni, 2016; Thompson et al., 2016). Secondly, the long‐term effect of beaver disturbances on wood‐boring beetle species richness may be noticeable mostly for late‐successional feeding guilds, which respond to strong fungal and bacterial attacks on well‐decayed deadwood (Esseen et al., 1992). Finally, it is possible that the long‐term effect of beavers on primary wood‐boring beetle species richness is mostly noticeable at the landscape scale rather than at the pond scale, especially in areas with many beaver ponds such as in our study area. Overall, some secondary deadwood dynamic mechanisms inherent to beaver ponds are possible and require further testing to assess events of fresh debris inputs that could affect primary wood‐boring organisms. Examples of such events would be pond recolonization from beavers (e.g., Hyvönen & Nummi, 2008), flash floods (e.g., Rosell et al., 2005), and windthrows (e.g., Franklin & Forman, 1987).

Although beetle species richness did not differ between site conditions, some compositional changes occurred. Despite apparent similar community structures (Figure 2a,b), the species differed in the rank they occupied in rank–abundance curves. In addition, 15 species were solely found around beaver ponds and 16 around control sites (Appendix). Beaver ponds may therefore act as a source habitat for species that will later disperse, causing a spill over that could increase landscape‐scale species richness. In fact, the impact of ecosystem engineers on species richness is said to be stronger when considering species requirements of engineered and unengineered habitat patches across the landscape (Hastings et al., 2007; Wright et al., 2002).

We already found evidence for the positive effect of the initial resource pulse from beaver activity on saproxylic beetle colonization and reproduction (Mourant et al., 2017). In this previous study, we found higher colonization from Scolytinae and Cerambycidae beetles around beaver ponds compared with unaltered watercourses by quantifying emergence holes on snags, which may be seen as a trace of the initial resource pulse. Here, we assessed the later effects of beaver disturbances on primary wood‐boring beetle activity. The fact that we can still detect primary wood‐boring beetle activity around old beaver ponds gives us insight on the strong attraction potential of newly created ponds. This could also be true with respect to the activity of other late‐successional feeding guilds (i.e., saprophagous, mycetophagous, and associated predators). In addition, snags closer to the disturbance area in the same studied ponds were the most heavily colonized by Scolytinae beetles (Mourant et al., 2017). Here, no general trends regarding the distance from the pond nor control sites have been identified (Figure 3), which highlights a more diffused effect of beaver activity compared with the actual colonization (see Gandiaga et al., 2018, for a discussion on visitation vs. colonization). The results of the present study together with our previous work in the same study system (Mourant et al., 2017) give a complementary view of the disturbance impacts at different time intervals, ranging from the initial pulse up to 44 years later. We suggest that the initial resource pulse has a far greater attractancy for primary wood‐boring beetles, yet the largest beaver disturbances have a higher probability to drive resident primary wood‐boring beetles over many years.

To conclude, we argue that beaver disturbances create dynamic habitats that positively affect primary wood‐boring beetles throughout vast time intervals. The initial flood and reflooding events can generate subsequent resource inputs for saproxylic beetles. Between reflooding events, primary wood‐boring beetles still benefit from large beaver‐disturbed sites, which highlights the unique characteristics of deadwood dynamics in these habitat patches. Further studies could assess the critical effects of beaver engineering on beetles across time to quantify the effect of the first flood on primary wood borers, as well as the succession of feeding guilds throughout the aging of the beaver‐disturbed site. Altogether, this adds beetles to the long list of organisms influenced by beaver disturbances, such as birds (e.g., Brown et al., 1996; Grover & Baldassarre, 1995; Gauvin et al., unpublished), mammals (e.g., Bailey & Stephens, 1951; Hawkes, 1973; Müller‐Schwarze, 1992), fish (e.g., Hägglund & Sjöberg, 1999; Murphy et al., 1989; Schlosser & Kallemeyn, 2000), benthic invertebrates (e.g., Margolis et al., 2001; McDowell & Naiman, 1986; Sprules, 1941), and reptiles and amphibians (e.g., France, 1997; Russell et al., 1999; Skelly & Freidenburg, 2000). The role of beavers in forest ecosystems by reaching multiple taxa at multiple spatiotemporal scales further exemplifies the need to study nontrophic interactions and their complex consequences in ecosystem management.

## CONFLICT OF INTEREST

None declared.

## AUTHOR CONTRIBUTIONS

**Alexandre Mourant:** Data curation (lead); formal analysis (supporting); investigation (lead); writing – original draft (lead). **Nicolas Lecomte:** Conceptualization (supporting); investigation (supporting); writing – review and editing (supporting). **Gaétan Moreau:** Conceptualization (equal); data curation (supporting); formal analysis (lead); funding acquisition (lead); investigation (supporting); methodology (lead); project administration (lead); writing – review and editing (supporting).

## Data Availability

Data are available on DRYAD at https://doi.org/10.5061/dryad.70rxwdbw9.

## Appendix

**TABLE A1:** Species, larval feeding guilds and abundance in each site conditions for specimens collected in the Kouchibouguac National Park, Canada in 2015–2016

Family	Species	Larval feeding guild^a^	Abundance in control sites	Abundance in beaver‐disturbed sites
Curculionidae; SF = Scolytinae	*Anisandrus sayi* (Hopkins, 1910)	Deciduous (2)	1,765	2,049
Curculionidae; SF = Scolytinae	*Polygraphus rufipennis* (Kirby, 1837)	Coniferous (2)	86	67
Curculionidae; SF = Scolytinae	*Dryocoetes autographus* (Ratzeburg, 1837)	Coniferous (2)	44	33
Curculionidae; SF = Scolytinae	*Xyleborinus saxeseni* (Ratzeburg, 1837)	Generalist (2)	32	32
Curculionidae; SF = Scolytinae	*Crypturgus borealis* Swaine, 1917	Coniferous (2)	27	16
Cerambycidae	*Evodinus Monticola Monticola* (Randall, 1838)	Coniferous (5)	25	17
Cerambycidae	*Pidonia ruficollis* (Say, 1824)	Deciduous (1)	20	9
Cerambycidae	*Strangalepta abbreviata* (Germar, 1824)	Generalist (5)	16	16
Curculionidae; SF = Scolytinae	*Trypodendron lineatum* (Olivier, 1795)	Generalist (2)	13	6
Cerambycidae	*Clytus ruricola* (Olivier, 1795)	Deciduous (5)	10	16
Curculionidae; SF = Scolytinae	*Pityophthorus* sp.	Coniferous (2)	7	22
Ptinidae	*Priobium sericeum* (Say, 1825)	Deciduous (3)	6	7
Curculionidae; SF = Cossoninae	*Rhyncolus macrops* Buchanan, 1946	Coniferous (1)	6	4
Cerambycidae	*Analeptura lineola* (Say, 1824)	Generalist (5)	5	38
Cerambycidae	*Idiopidonia pedalis* (LeConte, 1861)	Unknown (5,6)	3	0
Cerambycidae	*Monochamus scutellatus* (Say, 1824)	Coniferous (6)	3	1
Cerambycidae	*Pogonocherus Penicillatus* LeConte in Agassiz, 1850	Coniferous (1)	3	2
Cerambycidae	*Stictoleptura canadensis canadensis* (Olivier, 1795)	Coniferous (5)	3	15
Cerambycidae	*Xestoleptura tibialis* (LeConte, 1850)	Coniferous (6)	3	1
Curculionidae; SF = Cossoninae	*Carphonotus testaceus* Casey, 1892	Coniferous (1)	3	3
Curculionidae; SF = Scolytinae	*Dryocoetes affaber* (Mannerhiem, 1852)	Coniferous (2)	3	3
Ptinidae	*Ernobius luteipennis* LeConte, 1879	Coniferous (1)	3	0
Buprestidae	*Anthaxia inornata* (Randall, 1838)	Coniferous (7)	2	1
Cerambycidae	*Trachysida mutabilis* (Newman, 1841)	Deciduous (5)	2	7
Cerambycidae	*Rhagium inquisitor* (Linnaeus, 1758)	Coniferous (5)	2	1
Curculionidae; SF = Scolytinae	*Conophthorus* sp.	Coniferous (2)	2	1
Ptinidae	*Hemicoelus carinatus* (Say, 1823)	Deciduous (1)	2	3
Buprestidae	*Dicerca tenebrosa* (Kirby, 1837)	Coniferous (7)	1	0
Buprestidae	*Dicerca tuberculata* (Laporte & Gory, 1837)	Coniferous (7)	1	2
Cerambycidae	*Anthophylax viridis* LeConte, 1850	Deciduous (1)	1	0
Cerambycidae	*Astylopsis macula* (Say, 1826)	Deciduous (1)	1	5
Cerambycidae	*Clytus marginicollis* Laporte & Gory, 1835	Coniferous (1)	1	0
Cerambycidae	*Leptura subhamata* Randall, 1838	Coniferous (6)	1	0
Cerambycidae	*Pygoleptura nigrella nigrella* (Say, 1826)	Coniferous (1)	1	0
Cerambycidae	*Saperda obliqua* Say, 1826	Deciduous (5)	1	0
Curculionidae; SF = Scolytinae	*Dendroctonus punctatus* LeConte, 1868	Coniferous (2)	1	0
Curculionidae; SF = Scolytinae	*Dendroctonus simplex* LeConte, 1868	Coniferous (2)	1	0
Curculionidae; SF = Scolytinae	*Ips pini* (Say, 1826)	Coniferous (2)	1	0
Curculionidae; SF = Scolytinae	*Pityogenes hopkinsi* Swaine, 1915	Coniferous (2)	1	1
Curculionidae; SF = Scolytinae	*Pityokteines sparsus* (LeConte, 1868)	Coniferous (2)	1	4
Curculionidae; SF = Scolytinae	*Procryphalus mucronatus* (LeConte, 1879)	Deciduous (2)	1	0
Curculionidae; SF = Scolytinae	*Trypodendron betulae* Swaine, 1911	Deciduous (2)	1	0
Curculionidae; SF = Scolytinae	*Trypodendron rufitarsis* (Kirby, 1837)	Coniferous (2)	1	0
Lymexylidae	*Elateroides lugubris* (Say, 1835)	Deciduous (5)	1	0
Ptinidae	*Anobium punctatum* (DeGeer, 1774)	Generalist (4)	1	0
Ptinidae	*Ptilinus ruficornis* Say, 1823	Deciduous (3)	1	5
Cerambycidae	*Anthophylax attenuatus* (Halderman, 1847)	Generalist (5)	0	2
Cerambycidae	*Astylopsis sexguttata* (Say, 1826)	Coniferous (6)	0	1
Cerambycidae	*Judolia montivagans montivagans* (Couper, 1864)	Coniferous (5)	0	1
Cerambycidae	*Leptura plebeja* Randall, 1838	Coniferous (1)	0	1
Cerambycidae	*Oberea praelonga* Casey, 1913	Deciduous (1)	0	1
Cerambycidae	*Saperda candida* Fabricius, 1787	Deciduous (5)	0	1
Cerambycidae	*Tetropium cinnamopterum* Kirby in Richardson, 1837	Coniferous (1)	0	1
Cerambycidae	*Trachysida aspera brevifrons* (Howden, 1959)	Coniferous (1)	0	3
Cerambycidae	*Xylotrechus integer* (Haldeman, 1847)	Coniferous (1)	0	2
Curculionidae; SF = Conoderinae	*Acoptus suturalis* LeConte, 1876	Deciduous (1)	0	3
Curculionidae; SF = Cossoninae	*Phloeophagus apionides* Horn, 1873	Deciduous (1)	0	2
Curculionidae; SF = Scolytinae	*Dryocoetes caryi* Hopkins, 1915	Coniferous (2)	0	1
Curculionidae; SF = Scolytinae	*Xyloterinus politus* (Say, 1826)	Generalist (2)	0	1
Ptinidae	*Ernobius schedli* Brown, 1932	Coniferous (1)	0	1
Ptinidae	*Hemicoelus umbrosus* (Fall, 1905)	Coniferous (3)	0	1

Note

Identification was based on: Downie & Arnett, 1996; Bright, 1976; Arango & Young, 2012; Baker et al., 1970; Evans, 2014; Yanega, 1996; Bright, 1987.

^a^ From Dahlberg and Stokland (2004).
